# Change of the Guards

**DOI:** 10.3402/qhw.v10.27766

**Published:** 2015-03-06

**Authors:** Carina Berterö

Since Professor Lillemor Hallberg and Professor Karin Dahlberg started the *International Journal of Qualitative Studies on Health and Well-being (IJQHW)* in 2006 it has developed immensely, not least after 2010 when it became Open Access. With unhindered access to *all* content, readers in 172 countries have since then accessed the journal's website in their thousands, and more than 290,000 full-text articles have been downloaded – and most likely read, shared, cited and applied. The number of submissions has constantly increased over the years; today the rejection rate is over 50%. *IJQHW* is now indexed in PubMed/MEDLINE; CINAHL, Scopus, and PSYC-info to name a few of the most prestigious index providers. In addition it is stored in PubMed Central, the free full-text archive of biomedical and life sciences journal literature at the U.S. National Institutes of Health's National Library of Medicine (NIH/NLM). For three years in a row (2011–2013) the impact factor has steadily increased and is now 0.926; soon the 2014 impact factor will be here.

This is an incredible development! And so now that I have taken over as Editor-in-Chief I am both awed and excited. Some time ago, Lillemor Hallberg retired as Editor-in-Chief because of other priorities in life. After Karin Dahlberg left the Editorial team a couple of years ago, Lillemor Hallberg has been *primus motor* in developing *IJQHW* into the authoritative source of knowledge, data and information on qualitative studies that it is today. I want to thank both Lillemor Hallberg and Karin Dahlberg for their brilliant initiative to start *IJQHW*, and especially Lillemor Hallberg for all her hard work and professionalism to maintain the high standards that are so significant of the journal.

A few years back, due to the high inflow of manuscripts and the resulting increased work load, the editorial team was expanded with two Co-Editors, Professor Soly Erlandsson and myself. I am very pleased that Soly Erlandsson will continue to support me and the journal; with her professional integrity she is a true asset to the journal. To replace me as Co-Editor, I have appointed Associate Professor Ptlene Minick for her deep insight into qualitative research and long experience with various methods and theory building. I welcome her as a competent colleague and as my new collaborator in the development of *IJQHW*.

Ptlene Minick, PhD, is an Associate Professor in the Byrdine F. Lewis School of Nursing and Health Professions at Georgia State University, Atlanta, Georgia, in the United States. Dr. Minick began her program of research using qualitative methods during her doctoral studies. Graduating in 1992, her dissertation was entitled *The early recognition of patient problems in critical care*. Her work, using an interpretive methodology based on human experiences, resulted in theory generation. Throughout the years, she has had the opportunity to gain further expertise in qualitative methods focusing on patients in acute care and improving outcomes. Working to link nursing expertise with patient outcomes she extended her work to quantitative methods and used her early qualitative work as a framework for instrument development.

We are now in the process of renewing and revitalizing the Editorial Board. In view of the gradual internationalization of scientific publishing in general and of *IJQHW* in particular, we are aiming for a truly international board but also one which better reflects all the various disciplines that are represented in the journal. We will soon present the new board on the journal's website.

To sum up, I extend my sincere thanks to Lillemor Hallberg and Karin Dahlberg for having created a lasting forum for the exchange of facts, knowledge and ideas on qualitative research in relation to health and well-being; and especially to Lillemor Hallberg for her persistent work throughout the years. And I warmly welcome my colleague Ptlene Minick as new Co-Editor of the *International Journal of Qualitative Studies on Health and Well-being*.

*Carina Berterö* Editor-in-Chief

